# Oral *Salmonella msbB* Mutant as a Carrier for a *Salmonella*-Based Vaccine for Prevention and Reversal of Type 1 Diabetes

**DOI:** 10.3389/fimmu.2021.667897

**Published:** 2021-05-24

**Authors:** Jacob Cobb, Jeffrey Rawson, Nelson Gonzalez, Michael Hensel, Fouad Kandeel, Mohamed I. Husseiny

**Affiliations:** ^1^ Department of Translational Research & Cellular Therapeutics, Arthur Riggs Diabetes & Metabolism Research Institute, Beckman Research Institute, City of Hope National Medical Center, Duarte, CA, United States; ^2^ Division of Microbiology, University of Osnabrück, Osnabrück, Germany; ^3^ Faculty of Pharmacy, Zagazig University, Zagazig, Egypt

**Keywords:** type 1 diabetes, autoantigens, immunotherapy, immunomodulator, *Salmonella msbB* mutant, oral vaccination, diabetes reversal and prevention

## Abstract

A therapy that includes an oral vaccine for type 1 diabetes (T1D) using live attenuated *Salmonella* MvP728 (Δ*htrA/*Δ*purD*), cytokines (IL10 and TGFβ) and preproinsulin (PPI) antigen in combination with a sub-therapeutic dose of anti-CD3 mAb was developed by our team. The vaccine combination therapy reduced insulitis and prevented and reversed diabetes in non-obese diabetic (NOD) mice. Here, we show the effectiveness of an alternative *Salmonella* mutant (Δ*msbB*) as a carrier strain, which is anticipated to have lower risks of an inflammatory response and septicemia as a result of modification in the lipopolysaccharide (LPS) *via* detoxification of lipid A. This mutant strain proved to have highly reduced pathogenic side effects. *Salmonella* strain Δ*msbB* expressed autoantigens and in combination with cytokines and anti-CD3 mAb, successfully prevented and reversed T1D to levels comparable to the previously used carrier strain Δ*htrA/*Δ*purD*. Additionally, the *Salmonella msbB* mutant resulted in higher rates of host cell infection. These results further demonstrate the potential of an oral *Salmonella*-based combined therapy in the treatment of early T1D.

## Introduction

Oral delivery of antigens directly to the gut-associated lymphoid tissue (GALT) is an effective means of inducing tolerance (1–3). We have developed an oral *Salmonella*-based delivery system using the *Salmonella* Pathogenicity Island 2-encoded type III secretion system (SPI2-T3SS) effective in delivering an autoantigen (preproinsulin, PPI), in combination with plasmids encoding cytokines that are known to support the immune suppression (TGFβ and IL-10), to prevent and reverse type 1 diabetes (T1D) in non-obese diabetic (NOD) mice (4–6). The co-administration of a short-course of anti-CD3 monoclonal antibody along with a reduced dose of *Salmonella*-based vaccine and PPI/TGFβ/IL10 preserved insulin-positive cells, reduced insulitis and prevented/reversed T1D in NOD mice (5, 6).

The delivery system exploits *Salmonella*’s natural ability to survive and thrive in the vacuoles of GALT phagosomes (7–9). The *Salmonella*-containing vacuoles (SCV) within antigen-presenting cells (APCs) allow for self-propagation of the bacteria. From this location, the bacteria safely deliver a recombinant antigen into the host cell cytosol or deliver vectors that are expressed by the host cell directly, while bypassing the intestinal degradation (7, 9–11). This delayed delivery, or *in vivo* expression method utilizing the SPI2-T3SS (12, 13) increases the safety and efficacy of treatment by ensuring that the antigen expression only occurs after bacterial uptake by the APCs. The recombinant antigen is then processed and presented by the host APCs to other GALT cells, which then migrate to distant organs (14). The flexibility of the system allows rapid development of new immunotherapies which provides a robust delivery of multiple antigens in a safe and inexpensive manner. Such vaccines were very effective in eliciting CD8 and CD4 T cell-mediated immune responses in animal models of infection and cancer (7, 11, 15, 16).

A suitable *Salmonella* strain requires a balance between immune stimulation and safety. Wild type bacteria can cause fatal septicemia, yet overly attenuated bacteria will not stimulate the immune system. Previously, the double mutant strain MvP728 of *Salmonella* (Δ*htrA/*Δ*purD*) (4–7) was employed as a carrier. This strain was effective in the prevention and reversal of T1D in NOD mice (4–6). Yet there is some risk with this strain due to the presence of endotoxic lipopolysaccharide (LPS) in the cell envelope.

In Gram-negative bacteria like *Salmonella*, the LPS-containing cell envelope serves as a vital defense mechanism for *Salmonella* to survive in the host (17, 18). Modification of *Salmonella* LPS is a logical way to develop a safer vector (17, 19) by balancing the function with attenuation. Lipid A is a conserved LPS moiety composed of core oligosaccharide and acyl chains (20, 21). Wild-type LPS contains a mixture of hexa- and hepta-acylated lipid A, which strongly stimulates the Toll-like receptor-4 (TLR4) to induce an inflammatory response (22). Modifying the amount of lipid A acylation may limit its ability to activate TLR4. The *Salmonella* enzyme myristoyl transferase (MsbB) is known to modify LPS by adding myristic acid residues to lipid A, resulting in a hexa-acylated lipid (22). Lipid A is also modified by acyltransferase adding a palmitic acid to yield hepta-acylated lipid A (20). Mutating *msbB* resulted in one fewer acyl chains on lipid A and created a mixture of penta- and hexa-acylated lipid A. The Δ*msbB* altered the LPS, while maintaining an agonist effect on TLR4 induces weaker proinflammatory signaling than wild-type LPS (20, 21).

In this study *Salmonella* Δ*msbB* (18, 20, 22) virulence and its use as a potential delivery system for autoantigens was assessed as an alternative to the *Salmonella* double mutant Δ*htrA/*Δ*purD*. Also, the combination therapy of Δ*msbB* (PI+TGFβ+IL10) with anti-CD3 mAb to prevent and revert new-onset diabetes in NOD mice was tested.

## Materials and Methods

### Preparation of *Salmonella* Vaccines

The *Salmonella* strains and plasmids used in this study are listed in **Table 1**. The *msbB* deletion strain was generated as previously described (10, 23, 25) by λ Red-meditated deletion and insertion of an *aph* cassette using primers listed in **Table 2**. After confirmation of the resulting mutant strain, the *aph* cassette was deleted by flippase (FLP)-mediated site-specific recombination target (FRT) (26–28) to yield ST55 (Δ*msbB*, **Table 1**). The expression plasmids were prepared and transformed into an attenuated strain of *S. typhimurium* MvP728 (Δ*htrA/*Δ*purD*) (7), MvP1553 (Δ*rfaD*) and Δ*msbB* as described (4). Bacteria were cultured in Luria-Bertani (LB). If required for selection, ampicillin (100μg/ml), kanamycin and/or carbenicillin (50µg/ml) was added. The *Salmonella* mutant Δ*msbB* was cultured in LB media without sodium chloride as the strain is sensitive to high osmolarity (29). The mouse preproinsulin (mPPI) and mouse proinsulin (mPI) were expressed by the *Salmonella* SPI2-T3SS as fusion to effector protein (SseF) under the control of intracellular-activated promoter (P*_sseA_*) to mediate the translocation into host cell cytoplasm. The cytokine TGFβ and IL10 were expressed and secreted by host cells under the control of cytomegalovirus (CMV) immediate early promoter (4).

**Table 1:** Strains and plasmids employed.

Strains or plasmids	Relevant characteristics	Source or reference
***Salmonella*mutants**		
ST07 (MvP728)	Δ*htrA::FRT* Δ*purD*::FRT	(4–7)
ST06 (MvP1553)	Δ*rfaD*::FRT	(18)
ST49 (MvP1851)	Δ*msbB*::*aph*; Kan^R^	This study
ST55	Δ*msbB*::FRT	This study
**Plasmids**		
p2810	P*_sseA_ sscB sseF*::*lisA*::HA; Amp^R^ in pWSK29	(11)
pFPV25.1	GFP constitutive expression	(23, 24)
pMH 509	P*_sseA_ sscB sseF*::*mPPI*::mycDDK; Amp^R^ in pWSK29	(4)
pVAX1	P_CMV_ Kan^R^ mammalian expression plasmid	Invitrogen
mTGFβ	m*tgfβ1*::mycDDK; Kan^R^ in pCMV6	Origen
mIL10	m*il10*::mycDDK; Kan^R^ in pCMV6	Origen
pMH 524	P_CMV_ m*tgfβ*1, Kan^R^ in pVAX1	This study
pMH 525	P_CMV_ m*il10*; Kan^R^ in pVAX1	This study
pMH 531	P*_sseA_ sscB sseF*::GFP; Amp^R^ in pWSK29	This study
pMH 532	P*_sseA_ sscB* s*seF*::*mPI*::mycDDK; Amp^R^ in pWSK29	This study

Kan^R^, kanamycin resistance; Amp^R^, ampicillin resistance; FRT, FLP recombination target.

**Table 2:** Oligonucleotides employed.

Designation	Sequence (5´ to 3´)
GFP-EcoRV-For	GCAGATATCATGAGTAAAGGAGAAGAAC
GFP-XbaI-Rev	GGCTCTAGATTATTTGTATAGTTCATCCATGCC
mIL10-HindIII-For	CTGAAGCTTCCACCATGCCTGGCTCAG
mIL10-XhoI-Rev	GGTCTCGAGTCAGCTTTTCATTTTGATCATCATGTATG
TGFb-HindIII-For	CTGAAGCTTCCACCATGCCGCCCTC
TGFb-XhoI-Rev	CATCTCGAGTCAGCTGCACTTGCAGG
mTGFb-Check-For	TCCTGGCGTTACCTTGGTAAC
mTGFb-Check-Rev	CTGTCACAAGAGCAGTGAGC
mPI-HpaI-For	ATAGTTAACTTTGTCAAGCAGCACCTTTG
MycDDK-XbaI-Rev	TCGTCTAGATTAAACCTTATCGTCGTCATCCTTG
msbB-Del13-For	CCCTTTGCTGGCGACGCTGGGGCGTTTTGCCGGACGG
	CTGATTCCGGGGATCCGTCGACC
msbB-Del13-Red-Rev	AGCATAAAGCCTCTCTTACGAGAGGCTTTATTTATTTG
	ATTGTAGGCTGGAGCTGCTTCG
msbB-DelCheck-For	AATCCTTTCGCTATCCACAG
msbB-DelCheck-Rev	ATGAGCACCGTCGGTTCAAC

*Restriction enzyme sites are underlined.

### Plasmid Construction

All PCR amplifications were performed using a polymerase with proof-reading activity (Thermo Fisher Scientific, MA). Plasmid manipulations were performed according to standard procedures and the resulting plasmid constructs were confirmed by DNA sequencing (9, 23). The recombinant plasmids were transformed into DH5α *E. coli* TOP 10 competent cells (Invitrogen, CA) using standard procedure. Confirmed plasmid constructs were transformed into *Salmonella* strains by electroporation using the micro pulsar 2.5 kV, 200 Ω and 25 μF (Bio-Rad) (9, 23).

### Plasmid Construction of *gfp* Reporter

To test the expression level of SPI2-T3SS genes in different mutants of *S. typhimurium*, green fluorescent protein (GFP) was employed and expressed under promoter control of an operon in SPI2-T3SS of *Salmonella*. The DNA sequence encoding GFP was amplified from pFPV25.1 using the primers GFP-EcoRV-For/GFP-XbaI-Rev (**Table 2**). The *gfp* sequence was digested with EcoRV/XbaI and subcloned into EcoRV/XbaI digested p2810 resulting in pMH 531 (**Table 1**). In the plasmid pMH 531, the expression of *gfp* gene was under the control of the SPI2 promoter. GFP can then be produced as a fusion protein with SseF (11).

### Plasmid Construction of *tgfβ1* and *1l10* in pVAX1

Mouse cytokines TGFβ and IL10 were used as immunomodulators and were expressed by host cells under the control of the CMV promoter (4–6). The mammalian expression vector pVAX1 (Invitrogen, CA) was used to be consistent with the Food and Drug Administration (FDA) guidelines. For the construction of recombinant mouse TGFβ and mouse IL10 into pVAX1, PCR amplification of mouse *tgfβ1* and *il10* (from Origen, **Table 1**) sequences using the primers TGFb-HindII-For/TGFb-XhoI-Rev and mIL10-HindII-For/mIL10-XhoI-Rev was undertaken (**Table 2**). During the digestion with HindII/XhoI, the products were subcloned into HindII/XhoI that was digested by pVAX1 to produce pMH 524 and pMH 525 (**Table 1**).

### Plasmid Construction of Mouse Proinsulin

The ORF of mouse proinsulin (mPI) was amplified from pMH 509 (**Table 1**) (4) using the primers mPI-HpaI-For/MycDDK-XbaI-Rev (**Table 2)** and digested with HpaI/XbaI. This is followed by sub-cloning into plasmid p2810 that was digested with EcoRV/XbaI to produce plasmid pMH 532 (**Table 1**). The plasmid expression vector of mouse proinsulin was under the control of the SPI2 promoter P*_sseA_* and fused with SseF, which itself mediates the translocation to the host cell cytoplasm.

### Animal Vaccination

Seven-week-old female NOD/ShiLtJ mice were obtained from The Jackson Laboratory (Bar Harbor, ME) and received high-quality care at the animal facility at the City of Hope (COH) (4–6). The study was approved by the COH Institutional Animal Care and Use Committee (IACUC# 18017).

Normoglycemic or diabetic NOD mice were vaccinated by gavage as published (4–6) with 10^7^ CFU/mouse of *Salmonella* delivering plasmids for the expression of TGFβ+IL10 plus 10^5^ CFU/mouse of *Salmonella* expressing sseF-PPI. The total volume delivered bacteria per animal was 200µl of a 5% sodium bicarbonate solution. Animals have received the vaccine on days 0 and 7. Vehicle control mice were gavaged with 200µl of a 5% sodium bicarbonate solution. Mice were treated for 5 consecutive days i.p. (days -1 to 3 start of vaccination; 2.5 μg/mouse) with hamster anti-CD3 mAb as published (4–6). Blood glucose was measured every 3-4 days with a One Touch Ultra glucometer (LifeScan, Milpitas, CA). Mice were considered diabetic when two consecutive blood glucose values were more than 200mg/dL (11.1 mM).

### *In Vitro* Infection

Murine RAW264.7 macrophages were obtained from the American Type Culture Collection (ATCC no.-TIB-71) and maintained according to the provider’s instructions. Cultures were treated with *Salmonella* expressing GFP (pMH531) or *Salmonella* expressing mPPI (pMH509) at a multiplicity of infection of 10 for 25 min, washed, and cultured for 16–20 hours prior to expression analysis (11). The cells were immune-stained as previously described (7). Briefly, after infection, the cells were fixed with BD Cyofix/Cytoperm buffer at room temperature for 20 min and then washed 3 times with BD Cyofix/Cytoperm washing buffer. The slides containing *Salmonella* expressing mPPI were incubated with mouse anti-LPS of *S. typhimurium* O-4 (1E6) (Santa Cruz Biotechnology, CA, 1:50) and Guinea Pig anti-insulin (Abcam, 1:50) for 1 hour. Goat anti-mouse Alex-Flour-488 (Biolegend, 1/100) and anti-Guinea Pig-Cy3 (Abcam, 1/50) were used as secondary antibodies to detect *Salmonella* and PPI, respectively. The slides were mounted on VECTASHIELD antifade reagent containing DAPI (VECTOR Laboratories) and sealed with Nail polish (Sally Hansen). Fluorescent images were acquired on a Zeiss observer II fluorescence microscope equipped with a digital imaging camera (Zeiss AxioCam 506). Images were captured using ZEN Blue light software. *Salmonella* expressing GFP or PPI were quantified from 10 to 18 infected cells obtained from 5 to 8 fields using ImageJ software.

### Virulence and Fecal Shedding of Δ*msbB* Strain

Seven-week-old female NOD mice were orally administered with 10^5^, 10^7^, and 10^9^ CFU/mouse of the *Salmonella msbB* mutant. Mice were weighed every 48 hours and stool were collected every 48-72 h over 3 weeks. To analyze the shedding of *Salmonella* strains in vaccinated mice, approximately 100 mg of fresh stool was homogenized in 1 ml of PBS. LB agar plates deficient in sodium chloride and containing selective antibiotics were swabbed and the bacteria were characterized.

### Statistical Analysis

Kaplan-Meier survival curves were used to evaluate the incidence of diabetes between different groups of mice. The significance of the differences was determined by Mantel-Cox log-rank test analysis. For analysis of GFP- or PPI-expression between different *Salmonella* mutants, one-way ANOVA for unpaired values was used. All data were reported as mean ± SEM. Statistical calculations were performed using GraphPad Prism 9 software and a *p* value of <0.05 was considered significant.

## Results

### *In Vitro* Assessment of *Salmonella*-Based Expression

**Figure 1:**
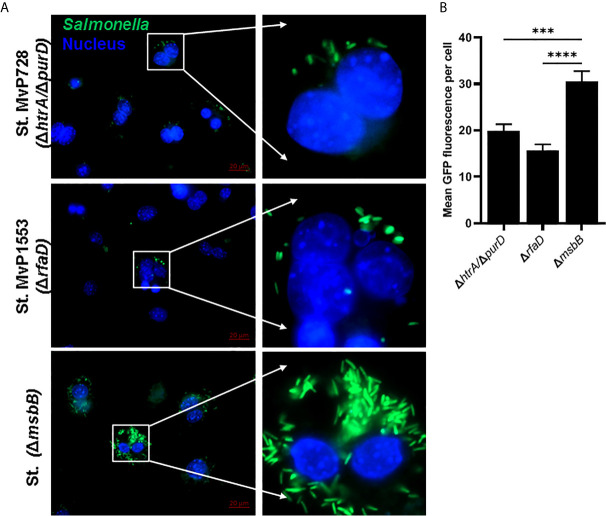
*In vitro* comparison of *Salmonella* SPI2-based protein expression in macrophage infected with different *Salmonella* mutants. RAW264.7 macrophages were infected with *Salmonella* mutants MvP728 (Δ*htrA*Δ*/purD*), MvP1553(Δ*rfaD*) or Δ*msbB* all of which were transformed with a plasmid encoding for GFP in frame with *sseF* under control of an intracellular promoter (pMH531, **Table 1**). Twenty hours after infection, cells were fixed and stained with DAPI for nuclei (blue) followed by analysis with Zeiss observer II fluorescence microscope to assess GFP within the host cells. Pictures were captured using ZEN Blue software. **(A)** A representative image (63X magnification, scale bar 20 µm) is shown. **(B)** Quantification of the mean fluorescence (green) of *Salmonella* expressing GFP per cell ± SEM. Data based on assessment of 15 to 18 infected cells in 5 to 8 fields. One-way ANOVA was used to determine significance (***p < 0.001 and ****p < 0.0001).

The expression of *Salmonella* SPI2-T3SS system as a vaccine carrier using *Salmonella* mutated in LPS such as MvP1553 *(*Δ*rfaD*) and Δ*msbB* along with the previously tested MvP728 (Δ*htrA/*Δ*purD*) was compared. All *Salmonella* mutants were transformed with a plasmid for expression of GFP under the control of SPI2 promoter and were used to infect RAW264.7 macrophages. After 20 hours of infection, significantly greater numbers of GFP-expressing *Salmonella* Δ*msbB* were noted in macrophages compared to GFP-expressing *Salmonella* Δ*rfaD* and Δ*htrA/purD* (**Figure 1A**). Quantification of GFP intensity per infected host cell indicated increased signal intensities for the Δ*msbB* strain compared to Δ*rfaD* and Δ*htrA/purD* strains (**Figure 1B**).

**Figure 2:**
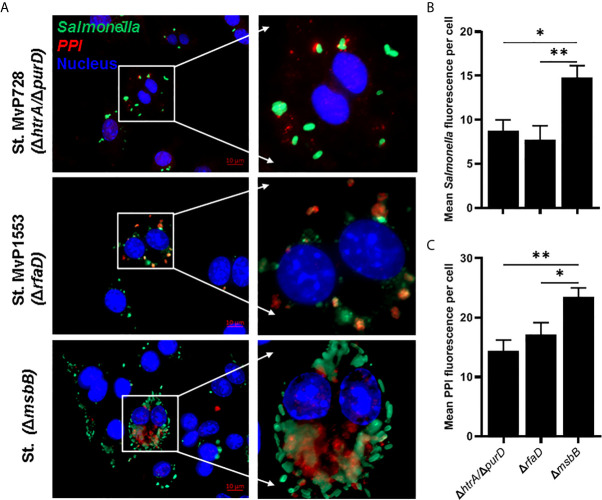
*In vitro* comparison of the translocation of PPI in a SPI2-T3SS-dependent manner between different *Salmonella* mutants. RAW264.7 macrophage cells were infected with *Salmonella* mutants MvP728 (Δ*htrA/*Δ*purD*), MvP1553(Δ*rfaD*) or Δ*msbB* harboring a plasmid encoding for mPPI in the frame with SseF, and effector protein of the SPI2-T3SS, and expressed under control of an intracellular promoter (pMH509, **Table 1**). After infection for 20 hours, cells were fixed and stained with Guinea Pig anti-insulin polyclonal and anti-*Salmonella* monoclonal primary antibodies. Secondary antibodies were applied for detection of insulin (red), *Salmonella* (green), and DAPI was used for staining of nuclei (blue) followed by analysis as in **Figure 1**. Cells were then visualized for the presence of bacteria located within the cytosol of host cells with colocalized red clusters of PPI. **(A)** A representative image (63X magnification, scale bar 10 µm) is displayed. **(B)** Quantification of the mean *Salmonella* fluorescence (green) per cell ± SEM. **(C)** Quantification of the mean PPI fluorescence (red) per cell ± SEM. *Salmonella* expressing PPI was quantified from 12 to 16 infected cells in 5 to 8 fields and results displayed are representative of 2 independent experiments. Statistical analysis using one-way ANOVA. The significance level is indicated by asterisks (*p < 0.05 and **p < 0.01).

To evaluate the expression and translocation of the foreign protein into the host cell cytosol *via* the SPI2-T3SS of *Salmonella*, murine macrophages were infected with different *Salmonella* mutants carrying the mPPI construct, namely a fusion protein of SPI2-T3SS effector SseF and mPPI. To examine whether the attenuation was altered the SPI2-T3SS-mediated expression and translocation, we used *Salmonella* mutants Δ*msbB* and Δ*rfaD* compared to Δ*htrA/*Δ*purD*. The mutant strain deficient in *msbB* translocated PPI into the cytosol of macrophages more than both *Salmonella* Δ*rfaD* and *Salmonella* Δ*htrA/*Δ*purD* (**Figure 2A**). After infection of macrophages, the mean green fluorescence (for *Salmonella*) and red fluorescence (for PPI) per cell was quantified (**Figures 2B, C**). A significant increase in the mean green and red fluorescence intensities was seen in the Δ*msbB* compared to the Δ*rfaD* and Δ*htrA/*Δ*purD* strains (**Supplementary Figure 1**).

### Virulence and Shedding of the *msbB* Mutant Strain

**Figure 3:**
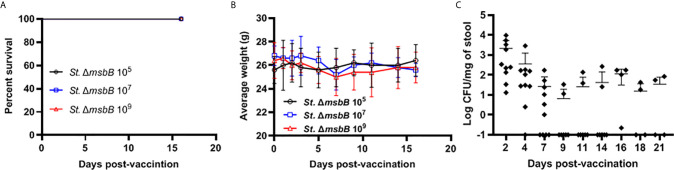
Virulence and shedding of *msbB* mutant strain. NOD mice were administered orally *Salmonella* Δ*msbB* at CFU values of 10^5^, 10^7^, or 10^9^ per mouse. **(A)** The survival of treated mice was followed for over 3 weeks post-vaccination (5 mice for each dose). **(B)** The side effects of Δ*msbB* were monitored by tracking the average of body weights for each group of mice over the time of the experiment. The data at each time point is represented the mean ± SD. **(C)** The presence of the Δ*msbB* in feces was determined over a period of 21 days (days 2, 4, 7, 9, 11, 14, 16, 18, and 21) post-vaccination. The means are indicated by horizontal bars ± SD (the number of mice ranged between 7-10 at each time point).

The *Salmonella* Δ*msbB* strain was used as an anti-cancer vaccine (30, 31). However, this strain was not previously studied for vaccination in diabetic NOD mice. We determined the safety of Δ*msbB* as a potential vaccine carrier. After oral administration of various doses of *Salmonella msbB* mutant, no mortality in NOD mice was observed. Mice tolerated up to 10^9^ CFU/mouse for three weeks post-vaccination (**Figure 3A**). Animals displayed no changes in their weights before and after vaccination (**Figure 3B** and **Supplementary Figure 2**). Additionally, no changes in the activity of treated mice were noted. Analysis of the fecal shedding indicated that the recombinant vaccine strain was present in the gastrointestinal tract for up to 3 weeks post-vaccination (**Figure 3C**).

### *Salmonella msbB* Mutant as a Vector for Prevention of Diabetes in NOD Mice

**Figure 4:**
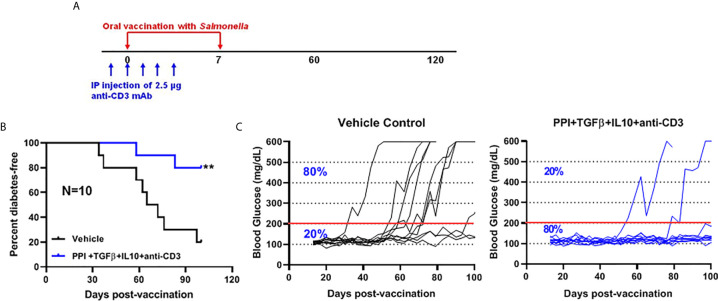
The oral *Salmonella msbB* mutant reduced the incidence of diabetes and prevented hyperglycemia. **(A)** NOD mice were vaccinated orally with a cocktail of *Salmonella* Δ*msbB* cultures transformed with plasmids encoding mPPI, mTGFβ, and mIL10 at days 0 and 7. Mice received anti-CD3 mAb for 5 consecutive days (-1 to 3). Blood glucose levels were measured every 3-4 days for 100 days. **(B)** Combined vaccine therapy using Δ*msbB* decreased the incidence of diabetes in NOD mice. Data is presented as the Log-rank plot of the percentage of NOD mice that remained diabetes-free. The difference between the group of mice vaccinated with combined therapy and the vehicle group was significant (**p < 0.01) by the log-rank (Mantel-Cox) test. **(C)** Combined vaccine using Δ*msbB* prevented hyperglycemia and stabilized blood glucose levels in NOD mice. N represents the number of the mice per treatment group. The red line indicates the threshold blood glucose level of 200 mg/dL.

We showed that two doses of *Salmonella*-based combined therapy (PPI+TGFβ+IL10) with a sub-therapeutic dose of anti-CD3 mAb prevented diabetes in NOD mice (4–6). The orally administered Δ*msbB* strain carrying both constructs for mPPI, mIL10, and/or mTGFβ was given in two doses at days 0 and 7 to normoglycemic NOD mice. Mice were injected with anti-CD3 antibody for five consecutive days (-1 to 3) (**Figure 4A**). Control mice were given the vehicle alone. Following administration, effects on blood glucose were assessed for 100 days (**Figure 4A**). A majority (80%) of the vaccinated NOD mice did not develop diabetes during the post-vaccination interval (**Figure 4B**) (Log-rank Mantel-Cox, p = 0.0064). In addition, the combination therapy-maintained blood glucose levels within a normal range in 80% of animals, compared with only 20% of animals given the vehicle (**Figure 4C**).

### *Salmonella*-Based Combination Therapy Using an Oral Live *msbB* Mutant Prevents Diabetes in NOD Mice

**Figure 5:**
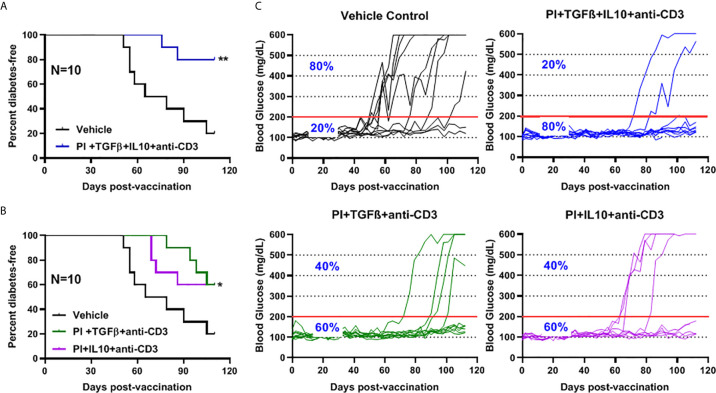
Combination therapy further reduced the incidence of diabetes and prevented hyperglycemia in NOD mice. NOD mice were orally vaccinated with a combination of *Salmonella* Δ*msbB* transformed with mPI+TGFβ+IL10, mPI+TGFβ or, mPI+IL10 and anti-CD3 mAb. Blood glucose levels were measured every 3-4 days for 100 days post-vaccination. **(A)** Combined vaccine therapy (mPI+TGFβ+IL10) decreased the incidence of diabetes in NOD mice compared to vehicle treated mice. **(B)** Combined vaccine therapy (mPI+TGFβ) or (mPI+IL10) decreased the incidence of diabetes in NOD mice compared to control mice. Data are presented as the Log-rank plot of the percentage of NOD mice that remained diabetes-free over the time course of the study. The difference between the group of mice vaccinated with combined therapy and the vehicle group was significant (*p < 0.05 and **p < 0.01) by the log-rank (Mantel-Cox) test. **(C)** Combined vaccine using *msbB* mutant prevented hyperglycemia and stabilized blood glucose levels in NOD mice. N represents the number of the mice per treatment group. The red line indicates the threshold blood glucose level of 200 mg/dL.

The role of combination therapy (PPI+TFGβ+IL10) with anti-CD3 for the prevention of diabetes in NOD mice, vaccinated with *Salmonella* Δ*htrA*Δ*/purD* mutant, was reported (5, 6, 32). Normoglycemic NOD mice were administered orally with *Salmonella* Δ*msbB* expressing autoantigen proinsulin (PI) combined with or without TGFβ and IL10 combined with a sub-therapeutic dose (2.5 mg/mouse) of anti-CD3 mAb. Animals administered with *Salmonella* Δ*msbB* along with the combination of PI+TGFβ+IL10 and anti-CD3 antibody were prevented from developing diabetes (Log-rank Mantel-Cox, p = 0.006) for more than 100 days post-treatment compared to control mice (**Figure 5A**). Interestingly, 60% of NOD mice were prevented from developing diabetes (Log-rank Mantel-Cox, p = 0.03) when treated with either PI+TGFβ or PI+IL10 plus anti-CD3 mAb compared to vehicle (**Figure 5B**). Further, orally administered *Salmonella* Δ*msbB* in combination with PI+TGFβ+IL10 and anti-CD3 mAb prevented the hyperglycemia and normalized blood glucose levels in 80% of mice for more than 100 days post-vaccination (**Figure 5C**) compared to 20% of mice, given vehicle alone, and 60% of the mice treated with either PI+TGFβ or PI+IL10 and anti-CD3 mAb (**Figure 5C**).

### *Salmonella msbB* Mutant as Therapeutic Vaccination Vector for Reversal of Diabetes in NOD Mice

**Figure 6:**
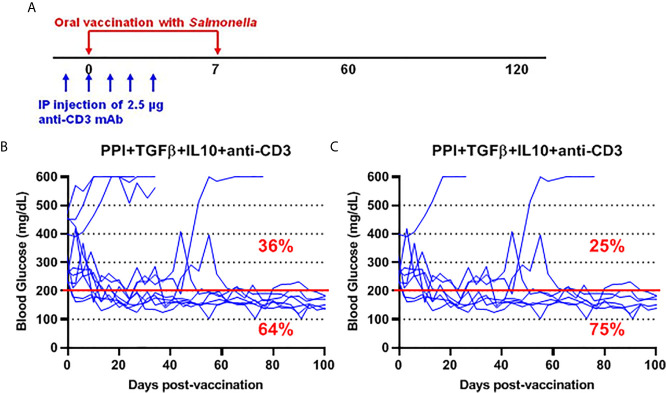
*Salmonella*-based combination therapy using the *msbB* mutant reversed diabetes in NOD mice. **(A)** New-onset diabetic NOD mice were vaccinated orally with a cocktail of Δ*msbB* transformed with plasmids encoding mPPI+mTGFβ+mIL10 at days 0 and 7. Mice received anti-CD3 mAb for 5 consecutive days at days (-1 to 3) post-vaccination. **(B)** Reversal of diabetes in mice after vaccination. **(C)** Reversal of diabetes in vaccinated mice with blood glucose less than 400 mg/dL. Data displayed as the blood glucose levels in NOD mice for 100 days. The red line indicates the threshold blood glucose level of 200 mg/dL.

To further investigate the safety and efficacy of the *msbB*, we assessed its potential to reverse diabetes. Early-stage diabetic NOD mice received the mutant vaccine carrying the PPI+TGFβ+IL10 constructs by gavage on days 0 and 7 and anti-CD3 mAb for five consecutive days (-1 to 3) post-vaccination (**Figure 6A**). Over 60% of vaccinated diabetic NOD mice that showed blood glucose levels < 200 mg/dL were persisted during the entire post-vaccination interval (**Figure 6B**). Interestingly, 75% of diabetic NOD mice with blood glucose levels less than 400 mg/dL were reversed to normal blood levels (less than 200 mg/dL) after vaccination (**Figure 6C**).

## Discussion

Although the *Salmonella* Δ*htrA/*Δ*purD* strain is attenuated, it retains outer membrane LPS that could, in theory, promote endotoxic shock. The goal of this study was to investigate the use of less toxic *Salmonella* strains as an oral *Salmonella*-based vaccine to prevent and revert diabetes in NOD mice. Our established *Salmonella*-based vaccine uses diabetic autoantigen expressed by the SPI2-T3SS construct inside APCs. It was not known how the SPI2-T3SS would function in *Salmonella* with mutated LPS like Δ*rfaD* and Δ*msbB*. We used GFP as a reporter protein that was expressed under control of a promoter in SPI2-T3SS of *Salmonella*. Elevated green fluorescence was noted in macrophages carrying the *Salmonella* Δ*msbB* which decreased for cells harboring the Δ*rfaD* and Δ*htrA/*Δ*purD* strains.

*Salmonella* Δ*msbB* was considered a potential therapeutic vaccine for cancer treatment (30, 31). *Salmonella* strain with a mutation in *msbB* (strain VNP20009) was used in a phase I trial in individuals with cancer (33). While the study did not show therapeutic effect, it did show the safety of the strain. The *msbB* mutation employed results in a modified version of LPS on the outer membrane that resulted in decreased TLR4 signaling and virulence *in vivo* (20, 22). Additionally, deletion of the *Salmonella msbB* gene resulted in decreased induction of TNFα when bacteria were administered to animals (20, 29).

Herein, we showed that a recombinant *Salmonella msbB* mutant combined with anti-CD3 mAb provided protection from diabetes in NOD mice. No side effects or unwanted reactions such as increased mortality, weight loss or change in activity was noted even after a high dose of the vaccine was given. However, we observed shedding of the *Salmonella* Δ*msbB* strain for at least 3 weeks. The *msbB* mutant was able to express and translocate high amounts of protein *in vitro*. Interestingly, large numbers of *Salmonella* Δ*msbB* were found inside infected cells and this was more than for MvP728 (Δ*htrA/*Δ*purD*) and MvP1553 (Δ*rfaD*). This finding suggests better survival and replication of the *msbB* mutant compared to other strains. Furthermore, the mutant bacteria delivered recombinant antigen into the host cell cytosol. The lesser effectiveness of the MvP1553 (Δ*rfaD*) mutant could be due to the high attenuation and/or the type of LPS modification, although this remains to be determined. The *rfaD* mutation affects assembly of the polymeric O-antigen of LPS and the mutant strains have a ‘rough’ phenotype. This results in loss of virulence due to fast clearance by antimicrobial effectors. This safety comes as the price of lower immune stimulation (18). In contrast, the Δ*msbB* strain possesses polymeric O-antigen but a detoxified LPS lipid A (18).

We observed fecal shedding of the Δ*msbB* strain for up to 9 days post-vaccination for most of the vaccinated mice. However, a small number of mice showed prolonged fecal shedding. This indicates the need for further attenuating mutations to ensure clearance of the vaccine. Examples of attenuating mutations include deletion of *lppAB* which encodes Braun’s lipoprotein (34) or the *purD* mutation present in VNP20009 (35). Another strategy for attenuation is based on conditional auxotrophy of the carrier strain (18). However, for therapeutic vaccines applied under close clinical supervision, less attenuation but efficient vaccine carrier strains can be considered as the strains can be controlled and eliminated by antibiosis.

The data herein indicated that the *msbB* mutant is suitable carrier for diabetes prevention (80%) in NOD mice. Still, enhanced infectivity may account for some of the better outcomes seen with the *msbB* mutant. Mice vaccinated with oral *Salmonella msbB* mutant showed persistence of the organisms for at least 3 weeks. This is long enough to allow expression and translocation of SPI2-T3SS effector proteins and presentation to immune cells (11). Interestingly, using mouse proinsulin (mPI) instead of mouse preproinsulin (mPPI) autoantigen in combination therapy prevents diabetes in 60% of vaccinated NOD mice compared to 20% in vehicle treated mice. The prevention of the incidence of diabetes is increased to 80% after vaccination with Δ*msbB* and PI+TGFβ+IL10+anti-CD3. This result highlights the importance of combination therapy for maximum effect.

Furthermore, the data demonstrate that the *Salmonella msbB* mutant reverted new onset diabetes (65%) in NOD mice. Interestingly, 75% of diabetic NOD mice with initial blood glucose levels less than 400 mg/dL demonstrated normal blood levels after vaccination. This suggests that combination therapy can reverse new onset diabetes when given after disease onset. Importantly, new onset disease is associated with the residual beta cell function. However, in instances of long-standing T1D there is a paucity of residual beta cell mass likely adversely impacting the vaccine effectiveness.

In summary, the *Salmonella* Δ*msbB* mutant is a potential vaccine for prevention and reversal of new onset diabetes. Further, the *Salmonella msbB* mutant poses less inflammatory risk. These results support the development of a *Salmonella msbB* mutant as a vaccine vector for clinical application.

## Data Availability Statement

The raw data supporting the conclusions of this article will be made available by the corresponding author, without undue reservation.

## Ethics Statement

The study was approved by the City of Hope Institutional Animal Care and Use Committee (IACUC# 18017).

## Author Contributions

MIH planned the experiments. MIH, JC, JR, and NG performed the experiments and collected data. MH generated plasmids and bacterial strains. JC, MIH, and FK analyzed the data. JC, MIH, and FK reviewed the data and wrote the manuscript. MIH and MH wrote and edited the submitted manuscript. MIH submitted the manuscript and was responsible for the accuracy of the data. All authors contributed to the article and approved the submitted version.

## Funding

This work was supported by the Wanek Family Project to Cure Type 1 Diabetes (award to MIH).

## Conflict of Interest

The authors declare that the research was conducted in the absence of any commercial or financial relationships that could be construed as a potential conflict of interest.

## References

[1] Cheminay C Hensel M. Rational Design of *Salmonella* Recombinant Vaccines. Int J Med Microbiol (2008) 298(1-2):87–98. 10.1016/j.ijmm.2007.08.006 17888730

[2] Holmgren J Czerkinsky C. Mucosal Immunity and Vaccines. Nat Med (2005) 11(4 Suppl):S45–53. 10.1038/nm1213 15812489

[3] Weiner HL da Cunha AP Quintana F Wu H. Oral Tolerance. Immunol Rev (2011) 241(1):241–59. 10.1111/j.1600-065X.2011.01017.x PMC3296283 21488901

[4] Husseiny MI Rawson J Kaye A Nair I Todorov I Hensel M. An Oral Vaccine for Type 1 Diabetes Based on Live Attenuated. Salmonella Vaccine (2014) 32(20):2300–7. 10.1016/j.vaccine.2014.02.070 24631074

[5] Mbongue JC Rawson J Garcia PA Gonzalez N Cobb J Kandeel F. Reversal of New Onset Type 1 Diabetes by Oral *Salmonella*-Based Combination Therapy and Mediated by Regulatory T-Cells in NOD Mice. Front Immunol (2019) 10:320. 10.3389/fimmu.2019.00320 30863412 PMC6400227

[6] Husseiny MI Du W Mbongue J Lenz A Rawson J Kandeel F. Factors Affecting *Salmonella*-based Combination Immunotherapy for Prevention of Type 1 Diabetes in non-Obese Diabetic Mice. Vaccine (2018) 36(52):8008–18. 10.1016/j.vaccine.2018.10.101 30416020

[7] Xiong G Husseiny MI Song L Erdreich-Epstein A Shackleford GM Seeger RC. Novel Cancer Vaccine Based on Genes of *Salmonella* Pathogenicity Island 2. Int J Cancer (2010) 126(11):2622–34. 10.1002/ijc.24957 PMC2993175 19824039

[8] Xu X Husseiny MI Goldwich A Hensel M. Efficacy of Intracellular Activated Promoters for Generation of *Salmonella*-based Vaccines. Infect Immun (2010) 78(11):4828–38. 10.1128/IAI.00298-10 PMC2976355 20732994

[9] Husseiny MI Hensel M. Evaluation of an Intracellular-Activated Promoter for the Generation of Live *Salmonella* Recombinant Vaccines. Vaccine (2005) 23(20):2580–90. 10.1016/j.vaccine.2004.11.035 15780440

[10] Husseiny MI Hensel M. Evaluation of *Salmonella* Live Vaccines With Chromosomal Expression Cassettes for Translocated Fusion Proteins. Vaccine (2009) 27(28):3780–7. 10.1016/j.vaccine.2009.03.053 19464562

[11] Husseiny MI Wartha F Hensel M. Recombinant Vaccines Based on Translocated Effector Proteins of *Salmonella* Pathogenicity Island 2. Vaccine (2007) 25(1):185–93. 10.1016/j.vaccine.2005.11.020 16887239

[12] Abrahams GL Hensel M. Manipulating Cellular Transport and Immune Responses: Dynamic Interactions Between Intracellular *Salmonella enterica* and its Host Cells. Cell Microbiol (2006) 8(5):728–37. 10.1111/j.1462-5822.2006.00706.x 16611223

[13] Hensel M. *Salmonella* Pathogenicity Island 2. Mol Microbiol (2000) 36(5):1015–23. 10.1046/j.1365-2958.2000.01935.x 10844687

[14] Coombes JL Powrie F. Dendritic Cells in Intestinal Immune Regulation. Nat Rev Immunol (2008) 8(6):435–46. 10.1038/nri2335 PMC2674208 18500229

[15] Evans DT Chen LM Gillis J Lin KC Harty B Mazzara GP. Mucosal Priming of Simian Immunodeficiency Virus-Specific Cytotoxic T-lymphocyte Responses in Rhesus Macaques by the *Salmonella* Type III Secretion Antigen Delivery System. J Virol (2003) 77(4):2400–9. 10.1128/jvi.77.4.2400-2409.2003 PMC141091 12551977

[16] Nishikawa H Sato E Briones G Chen LM Matsuo M Nagata Y. In Vivo Antigen Delivery by a *Salmonella typhimurium* Type III Secretion System for Therapeutic Cancer Vaccines. J Clin Invest (2006) 116(7):1946–54. 10.1172/JCI28045 PMC1481660 16794737

[17] Kong Q Six DA Roland KL Liu Q Gu L Reynolds CM. *Salmonella* Synthesizing 1-Dephosphorylated [Corrected] Lipopolysaccharide Exhibits Low Endotoxic Activity While Retaining its Immunogenicity. J Immunol (2011) 187(1):412–23. 10.4049/jimmunol.1100339 PMC3119770 21632711

[18] Frahm M Felgner S Kocijancic D Rohde M Hensel M Curtiss R 3rd. Efficiency of Conditionally Attenuated *Salmonella enterica* Serovar Typhimurium in Bacterium-Mediated Tumor Therapy. mBio (2015) 6(2):1–11. 10.1128/mBio.00254-15 PMC4453544 25873375

[19] Kong Q Yang J Liu Q Alamuri P Roland KL Curtiss R 3. Effect of Deletion of Genes Involved in Lipopolysaccharide Core and O-antigen Synthesis on Virulence and Immunogenicity of *Salmonella enterica* Serovar Typhimurium. Infect Immun (2011) 79(10):4227–39. 10.1128/IAI.05398-11 PMC3187260 21768282

[20] Khan SA Everest P Servos S Foxwell N Zahringer U Brade H. A Lethal Role for Lipid A in *Salmonella* Infections. Mol Microbiol (1998) 29(2):571–9. 10.1046/j.1365-2958.1998.00952.x 9720873

[21] Rietschel ET Kirikae T Schade FU Mamat U Schmidt G Loppnow H. Bacterial Endotoxin: Molecular Relationships of Structure to Activity and Function. FASEB J (1994) 8(2):217–25. 10.1096/fasebj.8.2.8119492 8119492

[22] Claes AK Steck N Schultz D Zahringer U Lipinski S Rosenstiel P. *Salmonella enterica* Serovar Typhimurium Delta *msbB* Triggers Exacerbated Inflammation in Nod2 Deficient Mice. PLoS One (2014) 9(11):e113645. 10.1371/journal.pone.0113645 25423082 PMC4244092

[23] Husseiny MI Hensel M. Rapid Method for the Construction of *Salmonella enterica* Serovar Typhimurium Vaccine Carrier Strains. Infect Immun (2005) 73(3):1598–605. 10.1128/IAI.73.3.1598-1605.2005 PMC1064926 15731059

[24] Valdivia RH Falkow S. Fluorescence-Based Isolation of Bacterial Genes Expressed Within Host Cells. Science (1997) 277(5334):2007–11. 10.1126/science.277.5334.2007 9302299

[25] Husseiny MI Hensel M. Construction of Highly Attenuated *Salmonella enterica* Serovar Typhimurium Live Vectors for Delivering Heterologous Antigens by Chromosomal Integration. Microbiol Res (2008) 163(6):605–15. 10.1016/j.micres.2006.10.003 19216101

[26] Datsenko KA Wanner BL. One-Step Inactivation of Chromosomal Genes in *Escherichia coli* K-12 Using PCR Products. Proc Natl Acad Sci U S A (2000) 97(12):6640–5. 10.1073/pnas.120163297 PMC18686 10829079

[27] Ellermeier CD Janakiraman A Slauch JM. Construction of Targeted Single Copy Lac Fusions Using Lambda Red and FLP-mediated Site-Specific Recombination in Bacteria. Gene (2002) 290(1-2):153–61. 10.1016/s0378-1119(02)00551-6 12062810

[28] Yamada Y Maeda M Alshahni MM Monod M Staib P Yamada T. Flippase (FLP) Recombinase-Mediated Marker Recycling in the Dermatophyte Arthroderma Vanbreuseghemii. Microbiol (Reading) (2014) 160(Pt 10):2122–35. 10.1099/mic.0.076562-0 24996827

[29] Low KB Ittensohn M Le T Platt J Sodi S Amoss M. Lipid A Mutant *Salmonella* With Suppressed Virulence and TNFalpha Induction Retain Tumor-Targeting *In Vivo* . Nat Biotechnol (1999) 17(1):37–41. 10.1038/5205 9920266

[30] Liu T Chopra AK. An Enteric Pathogen *Salmonella Enterica* Serovar Typhimurium Suppresses Tumor Growth by Downregulating CD44high and CD4T Regulatory (Treg) Cell Expression in Mice: The Critical Role of Lipopolysaccharide and Braun Lipoprotein in Modulating Tumor Growth. Cancer Gene Ther (2010) 17(2):97–108. 10.1038/cgt.2009.58 19713997 PMC2808459

[31] Kong Q Six DA Liu Q Gu L Roland KL Raetz CR. Palmitoylation State Impacts Induction of Innate and Acquired Immunity by the *Salmonella enterica* Serovar Typhimurium *msbB* Mutant. Infect Immun (2011) 79(12):5027–38. 10.1128/IAI.05524-11 PMC3232669 21930761

[32] Mbongue JC Alhoshani A Rawson J Garcia PA Gonzalez N Ferreri K. Tracking of an Oral *Salmonella*-Based Vaccine for Type 1 Diabetes in Non-obese Diabetic Mice. Front Immunol (2020) 11:712. 10.3389/fimmu.2020.00712 32411136 PMC7198770

[33] Toso JF Gill VJ Hwu P Marincola FM Restifo NP Schwartzentruber DJ. Phase I Study of the Intravenous Administration of Attenuated *Salmonella typhimurium* to Patients With Metastatic Melanoma. J Clin Oncol (2002) 20(1):142–52. 10.1200/JCO.2002.20.1.142 PMC2064865 11773163

[34] Erova TE Kirtley ML Fitts EC Ponnusamy D Baze WB Andersson JA. Protective Immunity Elicited by Oral Immunization of Mice With *Salmonella enterica* Serovar Typhimurium Braun Lipoprotein (Lpp) and Acetyltransferase (MsbB) Mutants. Front Cell Infect Microbiol (2016) 6:148. 10.3389/fcimb.2016.00148 27891321 PMC5103298

[35] Low KB Ittensohn M Luo X Zheng LM King I Pawelek JM. Construction of VNP20009: A Novel, Genetically Stable Antibiotic-Sensitive Strain of Tumor-Targeting *Salmonella* for Parenteral Administration in Humans. Methods Mol Med (2004) 90:47–60. 10.1385/1-59259-429-8:47 14657558

