# Supplementary material for: Oral Salmonella msbB Mutant as a Carrier for a Salmonella-Based Vaccine for Prevention and Reversal of Type 1 Diabetes
Source: Front Immunol. 2021 May 24;12:667897. doi: 10.3389/fimmu.2021.667897 (PMC8181392; doi:10.3389/fimmu.2021.667897)
Supplement: Supplementary file 1 [file DataSheet_1.pdf]

Oral *Salmonella msbB* mutant as a carrier for a *Salmonella*-based vaccine for prevention and reversal of type 1 diabetes

Jacob Cobb [1], Jeffrey Rawson [1], Nelson Gonzalez [1], Michael Hensel [2], Fouad Kandeel [1], and Mohamed I. Husseiny [1, 3,*]

[1] Department of Translational Research & Cellular Therapeutics, Diabetes & Metabolism Research Institute. Beckman Research Institute, City of Hope National Medical Center, Duarte, CA 91010-3000, USA.

[2] Division of Microbiology, CellNanOs – Center of Cellular Nanoanalytics Osnabrueck, University Osnabrueck, Osnabrueck, Germany.

[3] Faculty of Pharmacy, Zagazig University, Zagazig, Egypt

*Corresponding author:

Mohamed I. Husseiny
melsayed@coh.org

frontiers

Supplementary Fig. 1

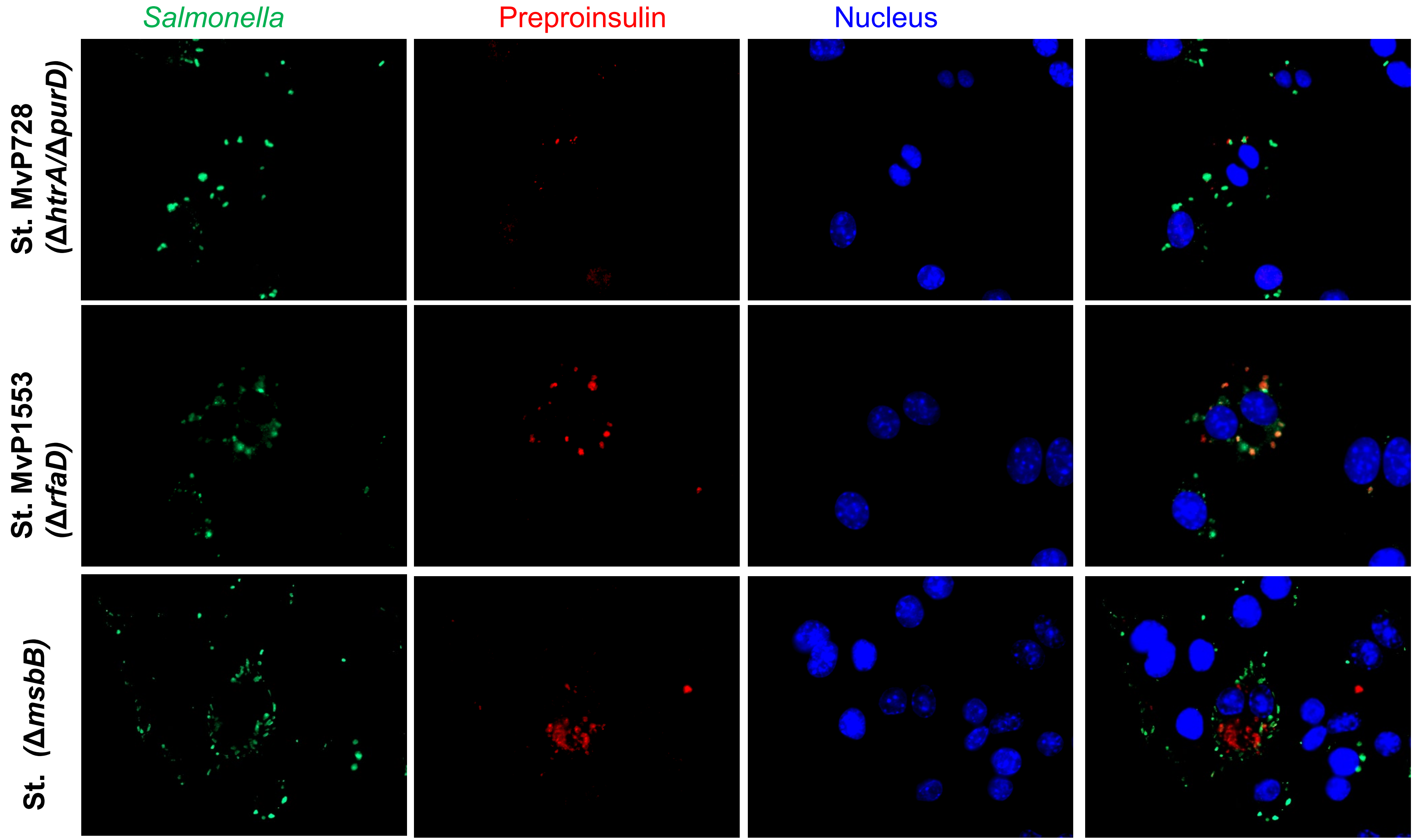

Suppl. Figure 1. SPI2-T3SS-dependent translocation of fusion proteins by intracellular *Salmonella*. Murine RAW264.7 macrophages were infected with various *Salmonella* mutants for the expression of fusion protein consisting of SseF-PPI (pMH 509). Twenty hrs after infection, the cells were fixed and processed for immuno-staining of *Salmonella* LPS (green), the PPI (red) and DAPI for nucleus (blue).

frontiers

Supplementary Fig. 2

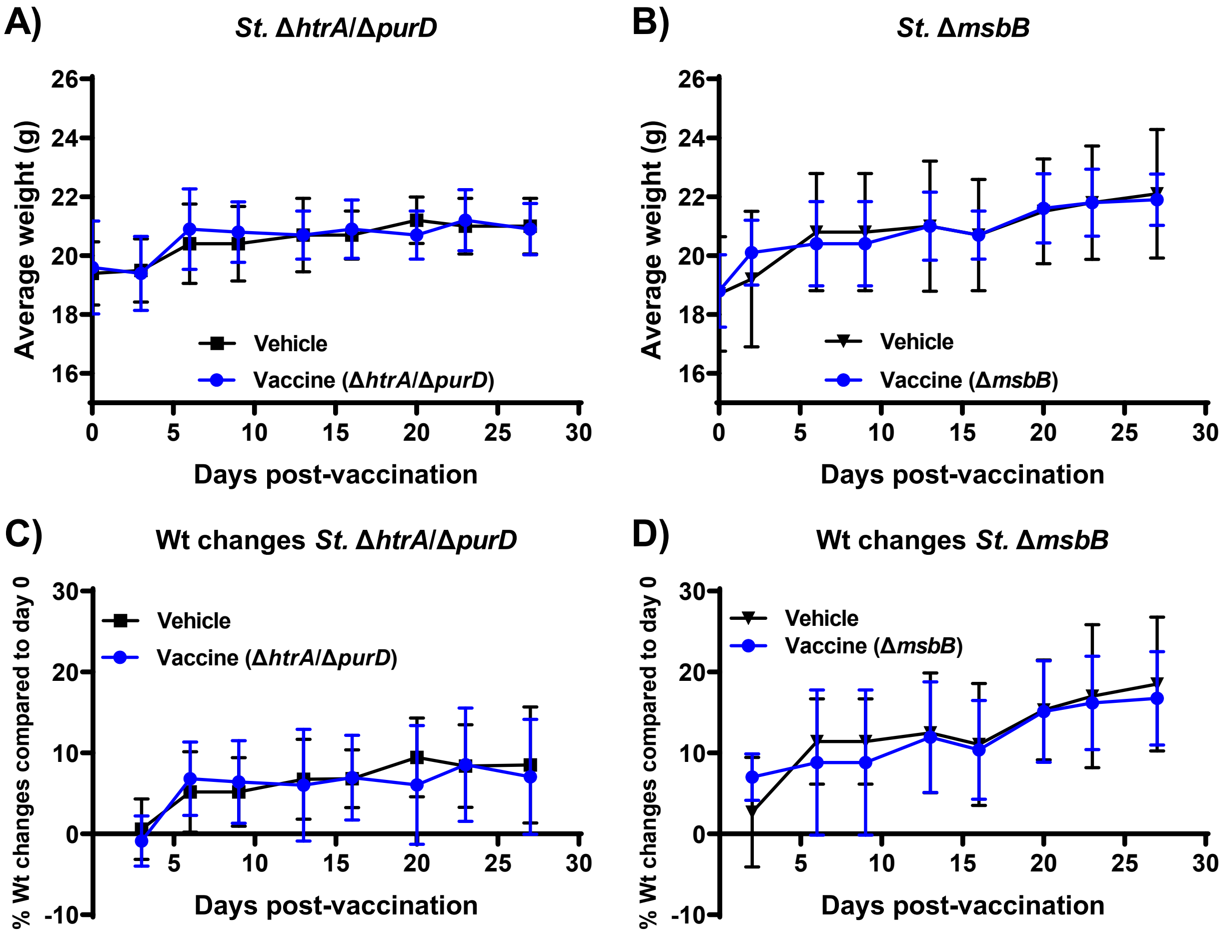

Supplementary Figure 2. Effect of a *Salmonella* mutant on the body weight of vaccinated mice. Female NOD mice (n=10) were vaccinated orally with the *Salmonella* based vaccine (PPI+TGF+IL10) combined with anti-CD3 mAb. Vehicle control mice (n=10) were orally gavaged with 200µl of a 5% sodium bicarbonate solution. No differences in the average body weights of mice receiving the Δ*htrA/* Δ*purD* **(A)** or Δ*msbB* **(B)** vaccine- and vehicle were noted. No differences between Δ*htrA/* Δ*purD* **(C)** or Δ*msbB* **(D)** vaccine- and vehicle-treated mice in the percentage of the gain or loss of body weight compared to day 0. Each time point represents the mean and ± SD of 10 samples. Statistical analysis using two-way ANOVA shows no significance between combined therapy and control group.
